# Population with Long-Term Care Needs in Six Latin American Countries: Estimation of Older Adults Who Need Help Performing ADLs

**DOI:** 10.3390/ijerph18157935

**Published:** 2021-07-27

**Authors:** Mauricio Matus-Lopez, Alexander Chaverri-Carvajal

**Affiliations:** Department of Economics, Quantitative Methods and Economic History, Universidad Pablo de Olavide, 41013 Seville, Spain; achacar@alu.upo.es

**Keywords:** long-term care, activities of daily living, frail elderly, Latin America, Uruguay, Chile, Argentina, Brazil, Colombia, Mexico

## Abstract

The population in Latin America is ageing, and there is an inevitable demand for long-term care services. However, there are no comparative analyses between Latin American countries of the dependency situation of older adults. This study aims to calculate and compare percentages of older adults who need help performing the activities of daily living in six Latin American nations. The study is observational, transversal, and cross-national and uses microdata drawn from national surveys conducted in Argentina (*n* = 3291), Brazil (*n* = 3903), Chile (n = 31,667), Colombia (*n* = 17,134), Mexico (*n* = 7909), and Uruguay (*n* = 4042). Comparable indicators of the need for help in performing the basic and instrumental activities of daily living were calculated. The percentages of older adults in need of help for basic activities of daily living ranged from 5.8% in Argentina to 11% in Brazil; for instrumental activities of daily living, from 13.8% in Mexico to 35.7% in Brazil; and combined, from 18.1% in Argentina to 37.1% in Brazil. Brazil thus has the highest indicators, followed by Colombia. The results warn of the frail physical condition of older people and the high potential demand for long-term care services. The information provided could be useful for further research on and planning for long-term care needs in Latin American and middle-income countries.

## 1. Introduction

One of the current main challenges for Latin American governments is to respond to the demands associated with the rapid and unprecedented ageing of the population—within the next 25 years, there will be more older adults (65 years and over) than children (under 15 years) [1]. This demographic change will require resources and reforms of social security systems [2], and both the World Health Organization and the Economic Commission for Latin America and the Caribbean have called on governments to develop long-term care (LTC) systems for older adults [3,4].

LTC encompasses the broad range of services and assistance provided to people who are limited in their ability to function independently on a daily basis, i.e., people who, for an extended period, need help to carry out activities of daily living (ADL), such as eating, showering, or getting dressed [5,6]. These services are classified as either home-based or residential; the former include services that allow the beneficiary to continue living in their own home (home care assistance, day and night care centers, telecare, etc.), while the latter are services provided in facilities, such as nursing homes, where the beneficiary has access to care and functional support 24 h a day—these are designed for the most severe health situations [7]. Internationally, the term most widely used to refer to these two types of services is ‘long-term care’, but in Spanish-speaking countries, the term ‘dependencia’ (dependency) is most often employed. The majority of LTC recipients are older adults: four out of five are over 65 years old [6].

During the 20th century, around 30 high-income countries, including Netherlands, Japan, and Spain, created their own LTC systems; in Latin America, Uruguay did so in 2015 and Costa Rica approved such a system in 2021 [8,9]. Many lessons can be learned from the experiences in other countries, one of which being that the first step in designing an LTC system is to know the number of people who will need it in the future [3,10]. Thus, the usual performance of basic and instrumental ADLs in a country’s physical and cultural context should be evaluated, together with other variables, such as the frequency and intensity of the help required by older people [11,12,13]. Each country can then determine the specific eligibility criteria for access to the services provided by their LTC systems [14,15,16,17,18].

Among Latin American countries, nationally representative estimates of the older population with LTC needs have been made in Uruguay, Chile, Argentina, Mexico, and Costa Rica. There are also studies based on less representative samples, such as in cities or administrative regions [19,20,21,22,23]. These estimates provide a wide range of results that vary in terms of the age threshold used for older adults (60 or 65 years old) and the estimation methodology [24,25,26,27,28]. With only one exception, these studies are not comparable between countries [29], and there are no nationally representative multi-country studies, so it is not possible to know the situation in one country with respect to the others.

This study aims to calculate and compare percentages of older adults who need help with ADLs in six Latin American countries. The methodology is based on the experiences of similar research in other countries, and the sources are national surveys conducted in Argentina, Brazil, Chile, Colombia, Mexico, and Uruguay, from which microdata have been drawn. The results are presented both raw and standardized according to population structure; they are thus comparable between countries.

## 2. Materials and Methods

### 2.1. Sources

The selection of countries was limited to Latin America and based on these two criteria: (a) the existence of a national survey on health, ageing or living conditions in which older adults aged 65 and over were represented and (b) the availability of the survey through public access or upon request to the institution that conducted it. 

Six Latin American countries were included: Argentina, Brazil, Chile, Colombia, Mexico, and Uruguay. These countries account for 70% of the population of the region. Despite similarities, the countries do differ; the highest per capita income of Chile and Uruguay, the largest population size of Brazil and Mexico, and the smallest population size of Uruguay can be highlighted. However, within the region, these are the countries that will have the largest older adult populations in the years to come (Table 1).

National surveys on health, ageing, and living conditions were used as sources for this observational and transversal study. There were four survey selection criteria: (a) examining ageing, health, or living conditions; (b) including a nationally representative sample of older adults; (c) analyzing variables related to ADLs; and (d) being the latest version released. In all the surveys, older adults were defined as people aged 65 and over. The description of the sources and the size of the sample (N) are shown in Table 2.

### 2.2. Variables

#### 2.2.1. Activities of Daily Living

Three groups of ADLs were used. The first group (basic ADLs, BADL) included basic activities taken from the Katz index [30]. Due to limitations of the surveys, the BADL group in the current study comprised only four such activities in each of the six countries: dressing, toileting, transferring, and feeding (Table 3, X: available in the survey; n.a: not available in the survey).

The second group (instrumental ADLs, IADL) was based on the instrumental activities of the Lawton scale [31]. Again, the group in the current study included only some of those activities because not all were assessed in the surveys. The IADL group comprised four instrumental activities—shopping, food preparation, medication, and handling of finances (Table 2)—but only in four countries because the surveys conducted in Chile and Uruguay did not provide this information.

Finally, we considered the ADL group as aggregation of the first and second groups: eight ADLs in total (four basic and four instrumental).

#### 2.2.2. Need for Help

The questionnaires of the six surveys included questions that assessed whether older adults needed help to perform ADLs, but the available responses varied. Argentina and Mexico used a binary option (yes/no), Brazil used three categories (does not need; needs but does not receive; needs and receives), Chile and Uruguay used scales to measure the frequency of need (never; almost never; sometimes; often; always) and Colombia used a combination of need for help and difficulty (no help and no difficulty; no help but difficulty; needs help; unable to do it). In the current study, the answers were coded as a binary variable called ‘help’, as shown in Table 4.

The information regarding the activities and questions considered is available in the original language in Appendix A.

### 2.3. Indexes

Three indexes were calculated. The first (BADL index) measures the percentage of older adults who require help from another person to perform at least one of the four activities in the BADL group; the second (IADL index) measures the percentage who need help performing at least one of the four activities in the IADL group; and the third (ADL index) measures the percentage who need help with any of the eight activities.

The indexes were calculated as follows:

$$Index=\frac{\sum P_{\left(x=1\right)}}{\sum \left[P_{\left(x=1\right)}+P_{\left(x=0\right)}\right]}$$

where *P* is the older adult population of the sample and *x* = 1 if they need help for any activity or *x* = 0 otherwise. The range of the index value is [0, 1]. The formula is valid for the three indexes when help is considered for BADLs, IADLs, or both, as appropriate.

### 2.4. Standardization

There are two reasons that may explain a country’s index (ADL or BADL) being higher than that of another country. First, the worse physical condition or health of its population. Second, a larger population who are either in the oldest age group (older adults are more dependent) or female (women are more dependent). Standardization consists of separating these effects. For this purpose, the same structure of population was applied to each country and the rates were weighted by age groups. Below is an example of the process for two countries and two age groups (Table 5).

Country X has a higher index (0.21) than country Y (0.20) because the population aged 80 years and over is larger in country X (40%) than country Y (20%). If the countries had the same age structure (standard structure), the index of country X (0.195 = 0.15 × 70% + 0.3 × 30%) would be lower than the index of country Y (0.225 = 0.15 × 70% + 0.4 × 30%).

The results are expressed in gross and standardized percentages. Standardization was performed based on the population structure of Latin America and the Caribbean, disaggregated by sex and age. The gross percentage shows the percentage of dependents, given the population structure of older adults of each country, by sex and age. The standardized percentage shows the same percentage if all countries had the same population structure.

### 2.5. Complementary Analyses

Three complementary analyses were performed. First, the saturation of responses was analyzed through the weighting of the three most frequent activities. We calculated the percentage of the index (BADL and IADL index) that would have been captured if only three activities had been considered. The higher the percentage, the lower the contribution of evaluating a fourth activity. Below is an example of the saturation of the BADL index for one country (Table 6).

The value of the index is 0.11. The contribution of each activity to the index is decreasing and is listed in descending order (column increment). Saturation measures the sum of the three first activities (90% = 0.095 ÷ 0.105 × 100). The higher the value, the less relevant the inclusion of a new activity and the more consistent the index.

The second analysis focused on association. Non-parametric Tau-b Kendall tests were performed for each country and activity. The higher the coefficient, the greater the association between the activities, for a given level of significance.

Finally, severity was calculated as the number of activities for which help was needed. The higher the value, the greater the percentage of people who require help in that number of activities.

## 3. Results

### 3.1. Need for Help

**Figure 1:**
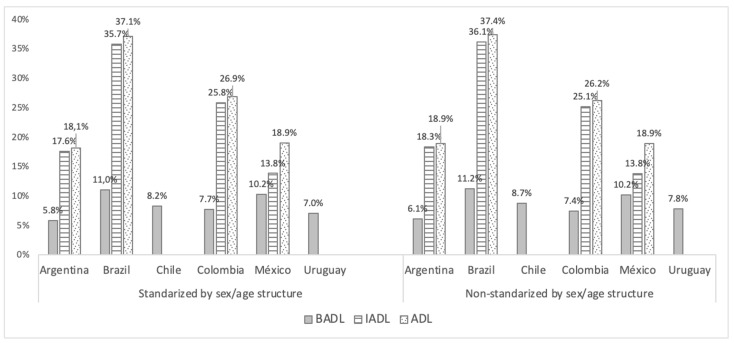
Percentages of older adults (65+) who needs help to perform BADLs, IADLs, or any of both (ADL) by country (standardized and non-standardized).

Figure 1 shows the percentages by country of older adults who need help with BADLs and IADLs. The percentages are presented both standardized according to sex and age structure and non-standardized.

The BADL index ranges from 5.8% to 11%. Argentina and Uruguay are the lowest, Colombia and Chile have intermediate values, and Mexico and Brazil both exceed 10%.

The range for the IADL index is wider, from 13.8% to 36.1%. Compared to the BADL results, the IADL index rates for Colombia are highest. In absolute terms, the rates for Brazil are the highest, followed by Colombia, with values close to 25%, then Argentina and Mexico. The consideration of all activities in the ADL index increased the rates for all countries, especially Mexico, which thus reaches the level of Argentina. Table 7 shows the results by sex and age.

The indexes confirm international trends in terms of age and sex. For instance, in the BADL index, the values increase with age; for women in the first age group (65–69 years), the values range between 2.1% for Argentina and 5.9% for Brazil, but for the older age group (80 years and over), Uruguay has the lowest percentage (15.8%) and Mexico the highest (29%). Among men, this age-related increase is not so evident, with values ranging from 2.4% for Colombia to 4.9% for Brazil in the lower age group and from 10.3% for Uruguay to 19.1% for Brazil in the higher age group.

In almost all age groups of age in the three indexes, women need more help than men, but there are exceptions, such as women and men aged 65–69 in Argentina and Chile, according to the BADL index. Nevertheless, in the 80-and-over age group, there is no inconsistency in the pattern of the indexes and countries: women always need more help than men. In these countries, the most notable exception to the pattern of gender and need for help is Colombia, where men have the highest total values for both the IADL and ADL indexes.

### 3.2. Complementary Analysis

The correlation between activities is high. All tests yielded significant and positive results, with stronger relationships among BADLs than IADLs (Table 8).

The saturation of three activities is particularly high; the three BADLs most frequently mentioned in the surveys were dressing, transferring, and walking, accounting for between 91% and 97% of all answers concerning BADLs. For IADLs, the three most frequently mentioned were shopping, food preparation and medication, which accounted for 92% to 97% of all answers.

The distribution of ADLs shows that approximately two out of every five people who gave positive answers need help for only one ADL, except in Chile, where the percentage is lower. A positive answer means that the person responded that they do need help (see Table 4). In contrast, more than 7% of the population in Argentina and Colombia responded that they require help with seven or more ADLs.

## 4. Discussion

The main objective of this study was to calculate and compare the percentages of older adults who need help in performing ADLs in six Latin American countries. The tools and definitions for calculating the need for help among older people vary between countries [32], but in both applied and research measures, indexes that comprise both BADLs and IADLs are often used [33,34,35], and most measures include the activities considered in the indexes of the current study [36]. Once a person starts needing help, the need often increases over time [37] and measuring the size of the population in need of such help allows the services provided by LTC systems—and their costs—to be planned [38].

In this study, we calculated and compared, for the first time, the proportions of older people who need help to perform ADLs in six Latin American countries. Comparisons between the results of this work and previous studies are not direct—as previously noted, measurement methodologies differ, making such comparison impossible. However, the results can be placed in perspective.

The results for Argentina are lower than those of previous studies that considered more activities [29], but the rates of help needed to perform ADLs were quite low regardless. In Brazil, most previous estimates examined the population aged 60 or over [27,39], and the rates calculated in the most recent works are closer to the rates in the current study [40].

Chile uses the least comparable methodology in the region. It applies an algorithm to different sources that allows different options for statuses, activities, and intensity. The estimates are therefore heterogeneous and cannot be compared with other countries [24,25,41,42]. This may explain why the results of the current research differ from those of most such studies, with values that are lower than the ones previously calculated.

On the other hand, the results of this study are consistent with estimates made for Colombia, although slightly lower because the government’s estimate considers a larger number of activities [43]. Most previous estimates for Mexico were based on an earlier source than the one used in this study and included more activities [44,45,46,47,48]; our results are therefore still high, though somewhat lower than in previous works. Uruguay is the only country with an existing LTC system, although its coverage is very limited. Nevertheless, its rates are the lowest, confirming the results of previous studies [17,18,49].

This research shows that the percentages of people aged 65 and more needing help to carry out ADLs in Latin America are higher than in high-income countries in Europe or Asia [13,35]. The calculations made for this study, in addition to shedding light on the social, economic, and political challenges associated with demographic change [39], reveal the regional demand for help and indicate the magnitude of the task involved in developing an LTC system [50].

The limitations of this study are related to the sources and the selection of activities. The microdata were drawn from national surveys that implemented different sampling methodologies, with both the language and wording of the questionnaires differing between countries. Despite these issues, this is the first time the LTC demands of older adults in Latin American countries have been compared, and the availability of sources for longitudinal information would help to confirm or reject the evidence presented in this study.

## 5. Conclusions

This research evaluates the percentages of older adults who need help in the performance of ADLs in six Latin American countries that account for 70% of the regional economy. The work undertakes a cross-national analysis for the first time, and the results obtained have not been published before.

The main important outcome is of methodological nature. The study proposes indexes based on other indicators that are commonly used in the field worldwide (Katz index and Lawton scale). The surveys conducted were different for each country, but the meaning of the questions was similar, and a methodological discussion is thus started regarding the possibilities for using our method to analyze other national measures.

The second important outcome relates to the results by country. The upper limits of the ranges in both indexes are almost double the lower limits, meaning that the differences between countries are significant. The highest values are found in Brazil, followed by Colombia, with Argentina and Uruguay having the lowest values. Standardization by sex and age shows that, at the same age and for the same sex, there are still important differences between countries. It will be interesting to further study the determinants of these results.

Complementary analyses also show consistency of results in terms of associations (correlation), representativeness of the activities (saturation), and concentration (distributions by number of ADL help).

The results have policy implications for the design and planning of national LTC systems. The region is in the process of discussing and implementing such services and requires evidence-based information on these issues, and the first step in the process consists of calculating the number of people who need help in performing ADLs [3,10]. Uruguay established its LTC system in 2015 [8,28], Costa Rica approved the development of its own system only in 2021 [9], and other countries are advancing in their national plans [26,27]. If the percentage of older adults needing help to perform ADLs is known and can be compared with other countries, the approximate size of the demand for care services can be calculated. This information will be useful for measuring the gap between supply and demand, and when combined with an estimate of the cost of the services, the economic resources required to implement these policies can be estimated. In addition, the disaggregation by sex and age allows the projection of future scenarios according to the demographic evolution of each country.

In summary, this study provides an international perspective of six Latin American countries. The high rates for all six are a cause for concern, and the implications of these findings are key to the development of LTC policies in the region, particularly for the purposes of determining demand, estimating the costs of policies, and planning the implementation of LTC systems.

## Figures and Tables

**Table 1:** Main indicators for countries selected; 2020 or latest year available.

Country	Population (Million)	% 65 and Older		GDP Per Capita	Country Group by Income	Life Expectancy
		2020	2050			
Argentina	45.61	9.5	15.1	9890.31	Upper-middle income	76.6
Brazil	213.99	8.4	20.5	8932.39	Upper-middle income	75.9
Chile	19.21	10.7	23.4	14,615.95	High-income	80.7
Colombia	51.27	8.2	19.1	6419.48	Upper-middle income	79.3
Mexico	130.26	7.0	15.0	10,024.42	Upper-middle income	76.0
Uruguay	3.49	12.2	19.0	17,681.21	High-income	77.0

**Table 2:** Description of the sources, country, name, year, coverage, and sample of adults 65 and older (Obs. and % sample) ^+^.

Country	Survey (Acronym and Year)	Coverage (by Age)	65+ Observations (%)
Argentina	Encuesta Nacional sobre Calidad de Vida de Adultos Mayores (ENCAVIAM, 2012)	60 years and older	3291 (70.8%)
Brazil	Estudo Longitudinal da Saúde dos Idosos (ELSI, 2015/16)	50 years and older	3903 (41.5%)
Chile	Encuesta de Caracterización Socioeconómica Nacional (CASEN, 2017)	All ages	31,667 (14.6%)
Colombia	Encuesta Nacional de Salud, Envejecimiento y Vejez (SABE, 2015)	60 years and older	17,134 (72.3%)
Mexico	Encuesta Nacional sobre Salud y Envejecimiento (ENASEM, 2018)	50 years and older	7909 (48.8%)
Uruguay	Encuesta Longitudinal de Protección Social (ELPS, 2015/16)	16 years and older	4042 (34.1%)

^+^ Sampling method: ENCAVIAM: probabilistic, random, clustered, multi-stage. ELSI: stratified, random, multi-stage. CASEN: random, geographic stratification, multi-stage. SABE: probabilistic, clustered, stratified, multi-stage. ENASEM: probabilistic, random, clustered. ELPS: random, stratified by clusters, multi-stage. See links to methodological documents in Appendix A.

**Table 3:** Activities of the Katz Index and activities of the Lawton scale included in the study, by country/survey.

Index/ Scale	Activities	Argentina ENCAVIAM	Brazil ELSI	Chile CASEN	Colombia SABE	Mexico ENASEM	Uruguay ELPS	Activities Selected
Katz Index	Bathing	X	X	X	X	X	n.a	No
	Continence	n.a	X	n.a	X	n.a	n.a	No
	Dressing	X	X	X	X	X	X	Yes
	Feeding	X	X	X	X	X	X	Yes
	Toileting	X	X	X	X	X	X	Yes
	Transferring	X	X	X	X	X	X	Yes
Lawton Scale	Using telephone	X	X	X	X	n.a	n.a	No
	Shopping	X	X	X	X	X	n.a	Yes
	Food preparation	X	X	n.a	X	X	n.a	Yes
	Housekeeping	X	X	X	n.a	n.a	X	No
	Transportation	X	X	n.a	X	n.a	n.a	No
	Medication	X	X	n.a	X	X	n.a	Yes
	Handling finances	X	X	n.a	X	X	n.a	Yes

**Table 4:** Reclassification of original variables into a new variable (‘help’) ^+^.

Variable Help/Country	Yes	No
Argentina (ENCAVIAM)	Yes	No
Brazil (ELSI)	Need but don’t get; Need and get	Don’t need
Chile (CASEN)	Almost never; Sometimes; Many times; Always	Never
Colombia (SABE)	Needs help; Can’t do it	No help and no difficulty; No help but difficulty
Mexico (ENASEM)	Yes	No
Uruguay (ELPS)	Almost never; Sometimes; Many times; Always	Never

^+^ Using conservative criteria, we categorized the missing values as ’no help required’ (No). The missing values were 0.2% or less in Brazil, Chile, and Colombia, 0% in Argentina and Uruguay, and 0.5% or less in Mexico.

**Table 5:** Standardization example (for two countries).

Percentage of Older Adult Population by Age	Population (%)		Index (Value)		Standard Structure
	Country X	Country Y	Country X	Country Y	
65–80	60%	80%	0.15	0.15	70%
80 and more	40%	20%	0.30	0.40	30%
Total, non-standardized	100%	100%	0.21	0.20	100%
Total, standardized			0.195	0.225	

**Table 6:** Saturation analysis example.

Need Help with…	Answer		Index		
	Yes	No	Value	Increase	Saturation
Only dressing	4.5%	95.5%	0.045	0.05	43%
Dressing and transferring	7.5%	92.5%	0.075	0.03	71%
Dressing, transferring and toileting	9.5%	90.5%	0.095	0.02	90%
Dressing, … and feeding	10.5%	89.5%	0.105	0.01	100%
Total	10.5%	89.5%	0.105		

**Table 7:** Percentages of older adults (65+) who needs help to perform any BADLs, IADLs, or both (ADLs) by sex, age, and country.

Index	Age	Argentina		Brazil		Chile		Colombia		Mexico		Uruguay	
		Men	Women	Men	Women	Men	Women	Men	Women	Men	Women	Men	Women
BADL index	65–69	3.1%	2.1%	4.9%	5.9%	3.5%	3.2%	2.4%	2.6%	2.9%	5.0%	3.0%	5.0%
	70–74	5.3%	2.9%	6.1%	11.2%	4.6%	4.8%	3.7%	2.8%	4.8%	8.4%	4.0%	5.8%
	75–79	4.5%	4.5%	8.0%	14.7%	6.5%	9.2%	7.9%	7.8%	7.2%	13.2%	6.2%	9.4%
	80+	11.4%	16.6%	19.1%	24.1%	17.2%	23.4%	15.9%	25.8%	17.7%	29.0%	10.3%	15.8%
	Total	5.6%	6.5%	8.6%	12.8%	7.1%	9.9%	6.2%	8.4%	7.5%	12.4%	5.5%	9.4%
IADL index	65–69	7.2%	9.9%	23.2%	26.4%	-	-	13.4%	8.4%	4.3%	7.8%	-	-
	70–74	11.3%	13.4%	25.0%	32.7%	-	-	19.6%	16.3%	5.5%	13.4%	-	-
	75–79	16.6%	27.1%	38.0%	42.6%	-	-	34.8%	26.9%	11.0%	18.8%	-	-
	80+	22.7%	42.2%	50.5%	62.5%	-	-	56.7%	57.4%	23.3%	34.5%	-	-
	Total	13.1%	22.1%	32.1%	38.7%	-	-	26.6%	23.9%	10.1%	16.9%	-	-
ADL index	65–69	7.4%	9.9%	24.2%	28.2%	-	-	14.4%	9.7%	6.2%	10.6%	-	-
	70–74	11.4%	15.4%	26.3%	33.7%	-	-	21.3%	17.2%	9.0%	17.2%	-	-
	75–79	17.6%	27.2%	38.3%	44.4%	-	-	35.4%	28.2%	14.0%	23.7%	-	-
	80+	24.2%	42.5%	52.5%	63.7%	-	-	57.6%	58.3%	32.5%	49.0%	-	-
	Total	13.7%	22.7%	33.2%	40.1%	-	-	27.7%	25.1%	14.3%	22.8%	-	-

BADL: Basic activities of daily living; IADLs: Instrumental activities of daily living; ADL: Activities of daily living.

**Table 8:** Complementary analysis.

Test/Indicator	Argentina	Brazil	Chile	Colombia	México	Uruguay
Correlation (Tau-b)						
Feeding &Toileting	0.534 **	0.519 **	0.642 **	0.460 **	0.537 **	0.505 **
Feeding & Transferring	0.552 **	0.493 **	0.603 **	0.398 **	0.551 **	0.427 **
Feeding & Dressing	0.550 **	0.397 **	0.575 **	0.473 **	0.230 **	0.486 **
Toileting & Transferring	0.695 **	0.678 **	0.808 **	0.694 **	0.709 **	0.599 **
Toileting & Dressing	0.632 **	0.554 **	0.770 **	0.641 **	0.331 **	0.701 **
Transferring & Dressing	0.702 **	0.544 **	0.806 **	0.576 **	0.365 **	0.607 **
Shopping & Food Preparation	0.624 **	0.451 **	-	0.582 **	0.587 **	-
Shopping & Medication	0.482 **	0.376 **	-	0.547 **	0.374 **	-
Shopping & Handle Finances	0.452 **	0.487 **	-	0.605 **	0.376 **	-
Food Preparation & Medication	0.530 **	0.372 **	-	0.518 **	0.414 **	-
Food Preparation & Handle Finances	0.485 **	0.436 **	-	0.462 **	0.413 **	-
Medication & Handle Finances	0.631 **	0.387 **	-	0.532 **	0.499 **	-
Saturation analysis (% cumulative activities)						
3 BADL over BADL Index	96.4%	97.5%	97.4%	93.8%	94.3%	91.3%
3 IADL over IADL Index	96.4%	92.6%	-	97.5%	96.6%	-
Distribution by number of ADL help (%)						
Only 1 ADL	43.6%	46.4%	27.0%	44.8%	39.7%	41.0%
2–4 ADL	37.9%	40.4%	73.0%	39.8%	49.8%	59.0%
5–6 ADL	10.7%	7.4%	-	7.6%	5.7%	-
7–8 ADL	7.8%	5.7%	-	7.8%	4.9%	-

**: Significant at the 0.05 level.

## Data Availability

Original data can be requested or downloaded from: http://datar.info/dataset/encaviam-2012 (ENCAVIAM 2012); http://elsi.cpqrr.fiocruz.br/ (ELSI 2015/16); http://observatorio.ministeriodesarrollosocial.gob.cl/encuesta-casen-2017 (CASEN 2017); https://www.minsalud.gov.co/salud/publica/epidemiologia/Paginas/Estudios-y-encuestas.aspx (SABE 2015); https://inegi.org.mx/programas/enasem/2018/?ps=microdatos (ENASEM 2018); https://www.elps.org.uy/ (ELPS 2015/6) (accessed on 20 July 2021).

## Appendix A

**Table A1:** BADL, IADL, and need of help. Original questions by survey (original languages).

Country (Survey)	Question (Original)	Activities	Answer
Argentina (ENCAVIAM) Methodological documents: https://bit.ly/36vPyJb	Le voy a mencionar algunas actividades para las que puede o no necesitar ayuda de otras personas. quisiera que me diga si necesita ayuda para realizar cada una de ellas. No tome en cuenta las limitaciones transitorias, que duren menos de tres meses. ¿Necesita ayuda de una persona para…	Comer en un tiempo razonable (cortar la comida, llenar los vasos, etc.) ^4^ Vestirse o desvestirse, incluyendo atarse los cordones ^1^ Bañarse, incluyendo entrar y salir de la ducha o bañera Peinarse, lavarse los dientes o lavarse la cara Andar de un lado a otro de su casa Usar el inodoro o higienizarse ^2^ Acostarse o levantarse de la cama ^3^ Andar de un lado a otro de su casa Subir y bajar escaleras Utilizar el teléfono, marcar los números y contestar una llamada Viajar en transporte público, taxis, remis, auto particular, etc. Organizar sus medicamentos y tomarlos ^7^ Manejar su dinero ^8^ Hacer las compras ^5^ Preparar comidas calientes ^6^ Hacer las tareas del hogar (lavar platos, tender camas, barrer, etc.)	1. Si 2. No Reclassification in HELP (dichotomous variable): No: No Yes: Si
Brazil (ELSI) Methodological documents: https://bit.ly/3k54Ix0	Agora farei algumas perguntas sobre atividades que algumas pessoas têm dificuldades para realizar, devido a problemas de saúde, incluindo sua memória. Peço que não considere dificuldades temporárias, aquelas que o(a) Sr.(a) espera que durem menos de três meses. O(A) Sr.(a) recebe ajuda para	Fazer sua higiene pessoal Preparar uma refeição quente ^6^ Administrar o próprio dinheiro ^8^ Utilizar algum tipo de transporte Fazer compras ^5^ Utilizar o telefone Administrar os próprios medicamentos^7^ Realizar tarefas domésticas leves (arrumar cama, tirar pó’, cuidar do lixo etc.) Realizar tarefas domésticas pesadas (lavar banheiro, limpar quintal, etc.) Caminhar um cômodo ou andar de um cômodo para outro no mesmo andar Vestirse ^1^ Tomar banho Comer ^4^ Deitar-se/ou levantar da cama ^3^ Usar o banheiro ^2^	1. Não, porque não precisa 2. Não, porque não ajuda 3. Sim Reclassification in HELP (dichotomous variable): No: Não, porque não precisa Yes: Sim; Não, porque não ajuda
Chile (CASEN) Methodological documents: https://bit.ly/3yZNkOL	Considerando sólo su estado de salud, ¿con qué frecuencia recibe ayuda de otra persona para:	Comer (incluyendo cortar comida y llenar los vasos) ^4^ Bañarse (incluyendo entrar y salir de la tina) Moverse /desplazarse dentro de la casa Utilizar el W.C. o retrete ^2^ Acostarse y levantarse de la cama ^3^ Vestirse ^1^ Salir a la calle Hacer compras o ir al médico Realizar sus tareas del hogar Hacer o recibir llamadas	1. Nunca; 2. Casi nunca; 3. Algunas veces; 4. Muchas veces; 5. Siempre. Reclassification in HELP (dichotomous variable): No: Nunca Yes: Casi nunca; Algunas veces; Muchas veces; Siempre
Colombia (SABE) Methodological documents: https://bit.ly/36wAFX4	Vamos a hablar de las actividades básicas de la vida diaria (BADL) que una persona puede realizar de forma independiente o dependiente. En cuanto a [actividad] Ud. fue capaz de…	Alimentación ^4^	1. Comer por usted mismo, comer Independiente 2. Necesitó ayuda de otra persona 3. Necesitó ser alimentado por otra persona
		Baño	1. Bañarse solo 2. Necesitó ayuda de otra persona para bañarse
		Vestido ^1^	1. Vestirse solo usted mismo 2. Necesitó que alguien lo ayudara 3. Lo tienen que vestir
		Arreglarse	1. Arreglarse sin ninguna ayuda 2. Necesita de la ayuda de otra persona
		Deposición	1. No tuvo accidentes (es continente) 2. Ha tenido accidentes ocasionales 3. No es capaz de retener (Incontinente)
		Micción	1. No tuvo accidentes (es continente) 2. Ha tenido accidentes ocasionales 3. No es capaz de retener (Incontinente)
		Uso del inodoro o sanitario ^2^	1. Ir solo al sanitario y utilizarlo 2. Necesitó algún tipo de ayuda cuando usó el sanitario 3. Requiere ayuda total cuando entra al sanitario
		Traslado cama-silla ^3^	1. Es capaz de pasarse solo de la cama a una silla 2. Necesita algún tipo de ayuda para pasarse de la cama a silla 3. No puede solo, necesita ayuda de otra persona 4. No es capaz de pasarse y necesita que lo carguen
		Deambulación	1. Camina solo sin problemas (Independiente) 2. Necesita algún tipo de ayuda para caminar 3. Está en silla de ruedas 4. No puede caminar (Inmóvil)
		Subir y bajar escalones	1. Es capaz de subir y bajar escaleras solo 2. Necesita ayuda para subir y bajar escaleras 3. No es capaz de subir ni bajar escaleras Reclassification in HELP (dichotomous variable): No: Camina solo sin problemas; Ir solo al sanitario y utilizarlo; Comer por usted mismo; Es capaz de pasarse solo de la cama a una silla; Vestirse solo usted mismo Yes: Resto de opciones
	Le mencionaré algunas actividades de la vida diaria, por favor responda teniendo en cuenta la que se ajuste a sus condiciones actuales. ¿Puede usted realizar las siguientes actividades? Es capaz	Maneja su propio dinero^8^ Es capaz de hacer las compras del diario (esp. comida) ^5^ Es capaz de preparar la comida ^6^ Es capaz de manejar sus propios medicamentos ^7^ Uso de trasporte publico o taxi Uso de teléfono	1. Lo hace sin ayuda de nadie y sin dificultad 2. Lo hace sin ayuda, pero con dificultad 3. Necesita (o necesitaría) ayuda para hacerlo 4. No es capaz de hacerlo Reclassification in HELP (dichotomous variable): No: Lo hace sin ayuda de nadie y sin dificultad Yes: Lo hace sin ayuda, pero con dificultad; Necesita (o necesitaría) ayuda para hacerlo; No es capaz de hacerlo.
Mexico (ENASEM) Methodological documents: https://bit.ly/36tZq6m	Por favor dígame si tiene alguna dificultad con cada una de las actividades que le voy a mencionar. Si usted no hace ninguna de las siguientes actividades, simplemente dígamelo. No incluya dificultades que cree que durarán menos de tres meses. (A quienes responden afirmativamente alguna de estas actividades, se le pregunta si recibe ayuda de alguien)	Vestirse, incluyendo ponerse los zapatos y los calcetines^1^ Caminar de un lado a otro de un cuarto Comer, por ejemplo, para cortar su comida ^4^ Acostarse y levantarse de la cama ^3^ Usar el excusado, incluyendo subirse y bajarse o ponerse en cuclillas ^2^ Preparar una comida caliente ^6^ Hacer compras de víveres/mandado ^5^ Tomar medicamentos (si toma alguno o tuviera que tomar alguno) ^7^ Manejar su dinero ^8^	1. Si 2. No Reclassification in HELP (dichotomous variable): No: No Yes: Si
Uruguay (ELPS) Methodological documents: https://bit.ly/2TZ43CL	¿Necesita ayuda de otras personas para realizar alguna de estas actividades?	Ir al baño (Incluye ubicación, manipular ropa, adoptar postura y limpiarse) ^2^ Peinarse, cortarse las uñas, lavarse el pelo o los dientes Comer o beber ^4^ Vestirse ^1^ Evitar riesgos de salud, pedir ayuda o seguir tratamiento Cambiar y mantener la posición ^3^ Desplazarse dentro del hogar Desplazarse fuera del hogar Realizar tareas domésticas (cocinar, hacer compras, limpiar) Participar en la vida social y comunitaria Comunicarse y tomar decisiones	1. No requiere 2. Casi nunca 3. Algunas veces 4. Muchas veces 5. Siempre Reclassification in HELP (dichotomous variable): No: No requiere Yes: Casi nunca; Algunas veces; Muchas veces; Siempre

^1,2,3,4^ Activities for BADL index; ^5,6,7,8^ Activities for IADL index.

**Table A2:** BADL, IADL and need of help. Original questions by survey (in English).

Country (Survey)	Question (Original)	Activities	Answer
Argentina (ENCAVIAM) Methodological documents: https://bit.ly/36vPyJb	I’m going to mention a few activities for which you may or may not need help from other people. I would like you to tell me if you need help in carrying out each of them. Do not take into account transitional limitations, which last less than three months. Need help from a person to...	Eating in a reasonable time (cutting food, filling glasses, etc.) ^4^ Dressing or undressing, including tying laces ^1^ Bathing, including getting in and out of the shower or bathtub Combing, brushing your teeth, or washing your face Walking around your house Using the toilet or sanitizing ^2^ Lying down or getting out of bed ^3^ Walking around your house Going up and down stairs Use the phone, dial numbers and answer a call Travel by public transport, taxis, remis, private car, etc. Organize your medications and take them ^7^ Managing your money ^8^ Shopping ^5^ Prepare hot meals ^6^ Doing household work (washing dishes, laying beds, sweeping, etc.)	1. Yes 2. No Reclassification in HELP (dichotomous variable): No: No Yes: Yes
Brazil (ELSI) Methodological documents: https://bit.ly/3k54Ix0	Now I’m going to ask you some questions about activities that some people have difficulty performing due to health problems, including their memory. I ask you not to consider temporary difficulties, those that you expect to last less than three months. You receive help to	Do your personal hygiene Preparing a hot meal ^6^ Managing your money ^8^ Use some kind of transport Shopping ^5^ Using your phone Administering your own medicines^7^ Perform light household work (make up bed, dust, take care of garbage, etc.) Perform heavy household work (bathroom wash, clean yard, etc.) Walk one room or walk from one room to another on the same floor Dressing ^1^ Showering Eating ^4^ Lie down/or get out of bed ^3^ Using the bathroom ^2^	1. No, because you don’t need 2. No, because it doesn’t help 3. Yes Reclassification in HELP (dichotomous variable): No: No, because you don’t need Yes: Yes; No, because it doesn’t help
Chile (CASEN) Methodological documents: https://bit.ly/3yZNkOL	Considering only your state of health, how often do you get help from someone else to:	Eating (including cutting food and filling glasses) ^4^ Bathing (including entering and exiting the tub) Move/move inside the house Use the W.C. or toilet ^2^ Lying down and getting out of bed ^3^ Dressing ^1^ Going out on the street Shopping or going to the doctor Perform your household chores Make or receive calls	1. Never; 2. Almost never; 3. Sometimes; 4. Many times; 5. Always. Reclassification in HELP (dichotomous variable): No: Never Yes: Almost never; Sometimes; Many times; always
Colombia (SABE) Methodological documents: https://bit.ly/36wAFX4	Let’s talk about the basic activities of daily living (BADL) that a person can perform independently or dependently. As for [activity] You were able to...	Eating ^4^	1. Eat for yourself, eat Independent 2. Needed help from someone else 3. Needed to be fed by someone else
		Bathing	1. Bathing alone 2. He needed help from someone else to bathe
		Dressing ^1^	1. Dress up only yourself 2. He needed someone to help him 3. They have to dress it
		Grooming	1. Fix yourself without any help 2. Need someone else’s help
		Deposition	1. Had no accidents (it’s continent) 2. Have had occasional accidents 3. Not able to retain (Incontinent)
		Urination	1. Had no accidents (it’s continent) 2. Have had occasional accidents 3. Not able to retain (Incontinent)
		Use of the toilet ^2^	1. Go alone to the toilet and use it 2. He needed some help when he used the toilet 3. Requires full help when you enter the toilet
		Transfer bed-chair ^3^	1. You are able to go alone from the bed to a chair 2. You need some help to move from bed to chair 3. You can’t alone, you need help from someone else 4. It is not able to pass and needs to be loaded
		Walking	1. Walk alone without problems (Independent) 2. You need some kind of walking help 3. You are in a wheelchair 4. Can’t walk (Motionless)
		Up and down steps	1. You are able to go up and down stairs alone 2. Need help going up and down stairs 3. Not able to go up or down stairs Reclassification in HELP (dichotomous variable): No: Walk alone without problems; Go alone to the toilet and use it; Eat for yourself; He is able to go alone from the bed to a chair; Dress up only yourself Yes: Other options
	I will mention some activities of daily living, please respond taking into account the one that fits your current conditions. Can you do the following activities? It is capable	Manage your own money ^8^ It is able to do the daily shopping (esp. food) ^5^ It is able to prepare food ^6^ You are able to manage your own medications^7^ Use of public transport or taxi Phone usage	1. It does it without help from anyone and without difficulty 2. It does it without help, but with difficulty 3. You need (or would need) help doing so 4. You’re not able to do it Reclassification in HELP (dichotomous variable): No: It does it without help from anyone and without difficulty Yes: He does it without help, but with difficulty; You need (or would need) help doing so; It is not capable of doing so.
Mexico (ENASEM) Methodological documents: https://bit.ly/36tZq6m	Please tell me if you have any difficulty with each of the activities I am going to mention. If you don’t do any of the following activities, just tell me. Don’t include difficulties that you think will last less than three months. (Those who answer in the affirmative are asked if they get help from anyone.)	Dressing, including putting on shoes and socks ^1^ Walking back and forth in a room Eating, for example, to cut your food ^4^ Lying down and getting out of bed ^3^ Use the toilet, including getting on and off or squatting ^2^ Prepare a hot meal ^6^ Shopping for groceries ^5^ Taking medicines (if you take any or need to take one)^7^ Managing your money ^8^	1. Yes 2. No Reclassification in HELP (dichotomous variable): No: No Yes: Yes
Uruguay (ELPS) Methodological documents: https://bit.ly/2TZ43CL	Do you need help from other people to do any of these activities?	Go to the bathroom (Includes location, handling clothes, posture and cleaning) ^2^ Combing, cutting nails, washing your hair or teeth Eating or drinking ^4^ Dressing ^1^ Avoid health risks, ask for help or follow treatment Change and maintain position ^3^ Moving within the home Moving outside the home Perform household chores (cooking, shopping, cleaning) Participate in social and community life Communicating and making decisions	1. Not required 2. Almost never 3. Sometimes 4. Many times 5. Always Reclassification in HELP (dichotomous variable): No: Not required Yes: Almost never; Sometimes; Many times; always

^1,2,3,4^ Activities for BADL index; ^5,6,7,8^ Activities for IADL index.
